# Association of chronic inflammation and accelerated atherosclerosis among an indigenous black population with chronic kidney disease

**DOI:** 10.1371/journal.pone.0232741

**Published:** 2020-07-10

**Authors:** Muzamil Olamide Hassan, Therese Dix-Peek, Raquel Duarte, Caroline Dickens, Sagren Naidoo, Ahmed Vachiat, Sacha Grinter, Pravin Manga, Saraladevi Naicker

**Affiliations:** 1 Divisions of Nephrology, Department of Internal Medicine, Faculty of Health Sciences, University of the Witwatersrand, Johannesburg, South Africa; 2 Internal Medicine Research Laboratory, Department of Internal Medicine, Faculty of Health Sciences, University of the Witwatersrand, Johannesburg, South Africa; 3 Division of Cardiology, Department of Internal Medicine, Faculty of Health Sciences, University of the Witwatersrand, Johannesburg, South Africa; 4 Department of Internal Medicine, Faculty of Health Sciences, University of the Witwatersrand, Johannesburg, South Africa; Nagoya University, JAPAN

## Abstract

**Introduction:**

Inflammation plays a major role in the development of atherosclerosis and cardiovascular morbidity and mortality in chronic kidney disease (CKD) patients. Toll-like receptor-4 (TLR4) is a major receptor for lipopolysaccharides (endotoxin) and other ligands involved in the pathogenesis of inflammation. We determined whether endotoxin levels and the presence of TLR4 polymorphisms are associated with markers of inflammation and atherosclerosis among South African CKD patients.

**Materials and methods:**

Endotoxin, lipopolysaccharide binding protein (LBP), serum CD14 (sCD14), interleukin-8 (IL-8), monocyte chemoattractant protein-1 (MCP-1) and carotid intima media thickness (CIMT) were measured in 160 participants (120 CKD patients and 40 controls). Associations between endotoxins and CIMT in the presence of sCD14, IL-8 and MCP-1, were assessed using odds ratios. Participants were screened for the presence of Asp299Gly and Thr399Ile TLR4 polymorphisms, and CIMT and inflammatory markers were compared between subjects with and without TLR4 polymorphisms.

**Results:**

Endotoxin levels correlated with sCD14 (r = 0.441, p<0.001) and MCP-1 (r = 0.388, p<0.001) levels while increased CIMT was associated with MCP-1 (r = 0.448, p<0.001), sCD14 levels (r = 0.476, p<0.001), LBP (r = 0.340, p<0.001), and IL-8 (r = 0.395, p<0.001). Atherosclerosis was associated with endotoxin levels (odds ratio: 4.95; 95% confidence interval: 2.52–9.73; p<0.001), and was predicted by higher serum levels of inflammatory markers. Analysis of patients with TLR4 polymorphisms showed reduced serum levels of inflammatory markers and CIMT values compared with the patients carrying the wild type TLR4 alleles.

**Conclusion:**

The study demonstrated associations between circulating endotoxaemia, systemic inflammation and accelerated atherosclerosis among South African CKD patients, and showed that the atherogenic predictive power of endotoxaemia was significantly increased by the presence of elevated levels of inflammatory markers. Additional findings, which must be confirmed, suggest that TLR4 polymorphisms are associated with low levels of inflammatory markers and CIMT values.

## Introduction

Atherosclerosis is increasingly being recognized as a chronic inflammatory condition and inflammatory mediators are thought to play a role in the pathogenesis of atherosclerosis [1–6]. Previous studies have shown that elevated endotoxin levels are associated with a risk of atherosclerosis [1, 7, 8]. Available evidence suggests that endotoxin may promote accelerated atherosclerosis through its ability to induce factors that play an important role in the endotoxin signalling pathway leading to a persistent chronic inflammatory state [9–11]. Circulating endotoxin binds to lipopolysaccharide binding protein (LBP), which facilitates binding of lipopolysaccharide (LPS) to soluble CD14 (sCD14) via toll-like receptor adaptor molecules, resulting in the downstream activation of nuclear factor- κB (NF-κB) and production of pro-inflammatory mediators [12–14].

Circulating endotoxaemia portends harmful outcomes both on cardiovascular function and structure, thus driving systemic inflammation, oxidative stress and atherogenesis [15]. Subclinical endotoxaemia significantly contributes to systemic inflammation, and thus, is a strong risk factor for atherosclerosis and cardiovascular disease [1]. Although, previous studies have evaluated the association of circulating endotoxaemia with accelerated atherosclerosis, none of these studies were carried out among indigenous black CKD patients with attendant high cardiovascular risk [2, 7].

Toll-like receptor-4 (TLR4) is a major receptor for LPS (endotoxin) and other ligands potentially involved in the pathogenesis of inflammation and vascular remodeling [16–20]. TLR4 is expressed by endothelial cells and monocytes, and its levels are markedly increased in atherosclerotic lesions, particularly in macrophages and endothelial cells [21, 22]. The responsiveness of an individual to LPS via the TLR4 signaling cascades provides an efficient innate immunity, which offers initial benefit but portends chronic vascular damage and increased risk of future atherosclerosis [16, 18].

Two common co-segregating missense mutations, A896G and C1196T resulting in the replacement of aspartic acid by glycine at amino acid position 299 (Asp299Gly) and the substitution of threonine by isoleucine at amino acid position 399 (Thr399Ile), respectively, have been identified in the human TLR4 gene [23]. These polymorphisms have been linked with endotoxin hypo-responsiveness and reduced risk of atherogenesis [13].

Although the TLR4 Asp299Gly polymorphism has been associated with reduced CIMT and lower serum levels of inflammatory cytokines in healthy populations, and reduced risk of myocardial infarction in Caucasians [16, 24, 25], to date, these studies have provided diverse results on the role of the TLR4 polymorphisms in the development of atherosclerosis [16, 24–30]. In addition, associations between TLR4 polymorphisms and susceptibility for atherosclerosis have not been previously investigated in CKD patients of African ancestry. In this study we determined the relationship between endotoxin-related inflammation and severity of atherosclerosis in South African CKD patients. Additionally, genotyping of TLR4 variants (Asp299Gly and Thr399Ile) was employed to investigate whether genetic variants of TLR4 were associated with reduced inflammatory response and susceptibility for atherosclerosis among indigenous black CKD cohorts.

## Materials and methods

The study included 120 CKD patients managed at the Charlotte Maxeke Johannesburg Academic Hospital (CMJAH). Forty age- and gender-matched black Africans who were staff and students at CMJAH were recruited as controls. Exclusion criteria included clinical signs of active or chronic infection, diabetes mellitus, seropositive status for hepatitis B, C and human immunodeficiency virus (HIV), autoimmune disease, liver dysfunction, malignancy, heart failure (New York Heart Association; NYHA III-IV) and use of anti-inflammatory or immunosuppressive therapy at least three months prior to enrolment. Information regarding age, race and tobacco smoking were documented. Blood pressure was recorded at the time of clinic visits in the arm with an Accusson mercury sphygmomanometer whilst the patient was in the sitting position. The average of three readings, 5 minutes apart, was taken as the blood pressure measurement. Mean arterial blood pressure (MABP) was calculated as diastolic blood pressure plus one third pulse pressure. Waist and hip circumferences were assessed using a tape measure. Waist-hip ratio (WHR) was calculated as waist measurement divided by hip measurement. This study was approved by the University of the Witwatersrand Human Research Ethics Committee (Protocol M130127).

### Blood sample collection and preparation

Blood for measurement of endotoxin and other inflammatory markers was collected into anticoagulant-free tubes after an overnight fast. Blood samples were allowed to clot at room temperature. Serum samples were separated within 30 minutes by centrifugation at 3000 rpm for 10 minutes, transferred into fresh polypropylene tubes and immediately frozen at −80ºC until ready for assay. Serum calcium, phosphate, creatinine and lipid profile were assayed using AdviaR 1800 auto-analyzers (Siemens Healthcare Diagnostics Inc, USA).

### Endotoxin levels

Using previously described methods [31], serum endotoxin was quantified using the Limulus amebocyte lysate QCL-1000^TM^ assay (Lonza Walkersville, USA) according to manufacturer’s instructions.

### Lipopolysaccharide binding protein concentrations

Serum levels of LBP were measured using a commercial human LBP ELISA kit, Hycult HK315 (Hycult biotechnology, Uden, the Nertherlands) according to manufacturer’s protocol. Absorbance was measured using an ELx800 Universal plate reader (BioTek Instruments, Inc, VT, USA).

### Serum sCD14, IL-8 and MCP-1 concentrations

Serum sCD14, IL-8 and MCP-1 assays were analyzed using Luminex® Performance Assay multiplex kits (R&D Systems, Inc. Minneapolis, USA). Assays were in accordance with the manufacturer’s instructions. Data were acquired on the Bio-Plex^TM^ 200 system (Bio-Rad, Texas, USA). Fluorescence intensity for MCP-1, IL-8 and sCD14 were read in the bead region 53, 54 and 59 respectively, and concentrations generated automatically using Bio-Plex manager software, version 5.0 (Bio-Rad Laboratories Inc, Hercules, USA).

### DNA extraction and TLR4 genotyping

Genomic DNA was extracted from whole blood using a modified salting out method [32] and the concentrations determined using a NanoDrop™ spectrophotometer (Thermo Scientific, Massachusetts, USA). The TLR4 Asp299Gly and Thr399Ile polymorphisms were determined by utilizing polymerase chain reaction (PCR) which was performed for amplification using optimised primers sets (TLR4-299; Forward: 5’-GAT TAG CAT ACT TAG ACT ACT ACC TCC ATG-3’, Reverse: 5’-GAT CAA CTT CTG AAA AAG CAT TCC CAC-3’ and TLR4-399; Forward: 5’-GGT TGC TGT TCT CAA AGT GAT TTT GGG AGA A-3’, Reverse: 5’-ACC TGA AGA CTG GAG AGT GAG TTA AAT GCT-3’). The PCR was performed using KAPA2G Robust HotStart Ready Mix PCR Kit (Kapa Biosystems, Massachusetts, USA) as per the manufacturer’s protocol. The PCR was run on a thermocycler (MJ Mini Thermal cycler, Bio-Rad, USA) according to manufacturer’s instructions. The PCR conditions were 3 mins of denaturation at 94°C, followed by 40 cycles (95°C for 15 secs, 60°C for 15 secs, and 72°C for 30 secs), and finally 72°C for 60 secs. The PCR products for Asp299Gly and Thr399Ile alleles were digested with restriction enzymes *NcoI* (5’-C/CATGG-3’) and *HinfI* (5’-G/ANTC-3’) respectively [33]. All the PCR products were visualized on a 2% agarose gel stained with ethidium bromide, with the aid of an image analyser (Gel Doc^TM^ EZ Imager, Bio-Rad, USA). All genotypes were assigned by independent investigators who were blinded to the results of CIMT measurements and markers of immune activation of the participants.

### Carotid intima media thickness

Carotid intima media thickness was assessed using high resolution B-mode ultrasonography with the aid of a L3-11 MHz linear array transducer (Philips Corporation USA) as previously described [34]. The CIMT was measured in plaque-free areas and all measurements were performed by the same sonographer who was blinded to the clinical details, laboratory data and results from the genetic analyses of the participants.

### Data analysis

Data analyses were performed using the statistical package for social sciences (SPSS) 16 (SPSS, Inc., Chicago IL). Data were presented as means ± SD or medians and interquartile ranges (IQR), where appropriate. Categorical data were compared using chi-square test and continuous data using student *t*-test or Mann-Whitney test. Correlation between continuous variables was examined by the Spearman’s rank correlation coefficient. Further analysis was performed after categorising endotoxin levels into two groups (cut-off value of 0.5 EU/ml). The categorisation of endotoxin levels (≤ 0.5 EU/ml and > 0.5 EU/ml) was adopted from a previous study [2]. In addition, CIMT measurements, sCD14, IL-8 and MCP-1 concentrations were subdivided into two groups according to the median values. Subclinical atherosclerosis was defined as CIMT > 0.55 mm. Association between endotoxin and atherosclerosis was assessed using logistic regression analysis.

To determine whether the baseline IL-8 or MCP-1 or sCD14 levels were an effect modifier of the relationship of circulating endotoxin to subclinical atherosclerosis, we performed a stratified multivariable logistic regression analysis, aimed at estimating the atherogenic predictive power of endotoxin in the presence of immune mediators, including IL-8, MCP-1 and sCD14. The models were adjusted for age, blood pressure, smoking, eGFR and cholesterol. Genotype frequencies were compared using Fisher’s exact test. A Mann-Whitney test was used to compare levels of markers of inflammation between patients with wild-type TLR4 and those with Asp299Gly allele alone, as well as those with combined TLR4 mutant alleles. A p-value <0.05 was regarded as statistically significant.

## Results

The patients’ characteristics and laboratory data are summarized in Table 1. At baseline, 22.5% and 23.3% of the CKD patients received angiotensin-II receptor blockers/angiotensin-converting enzyme inhibitors and statins, respectively. Endotoxin concentrations in CKD patients were significantly higher than that of the controls (p<0.001). Carotid intima media thickness was significantly higher among CKD patients compared to the controls (p<0.001). There were no significant differences in the CIMT values between patients who were treated with ACEI/ARB/statins and those who did not received the treatment [ACEI/ARB: 0.56 (0.49–0.63) vs 0.56 (0.50–0.65), p = 0.566; statins: 0.58 (0.52–0.68) vs 0.55 (0.49–0.63), p = 0.154]. According to the American Society of Echocardiography, 25% of CKD patients had high CVD risk (CIMT values ≥75^th^ percentile), 46% of patients had average risk for CVD (CIMT values in the 25th to 75th percentile), while 29% of patients presented with CIMT values lower than 25^th^ percentile, considered lower CVD risk.

**Table 1:** Demographic, clinical and laboratory data of the study population.

Parameter	Patients (N = 120)	Control (N = 40)	P value
Age (years; mean ± SD)	41.1 ± 10.2	42.2 ± 10.1	0.573
Sex (Male/Female)	64/56	22/18	0.709
Smoking (Yes/No)	17/103	2/38	0.121
MABP (mmHg)	134.8 (118.8–150.0)	117.0 (107.8–130.6)	<0.001
Waist-Hip ratio	0.91 (0.87–0.96)	0.88 (0.83–0.93)	0.122
eGFR (ml/min/1.73m^2^)	9.0 (4.0–44.0)	96.5 (83.0–119.0)	<0.001
Total cholesterol (mmol/L)	4.20 (3.43–5.18)	4.00 (3.33–4.88)	0.519
HDL (mmol/L)	1.20 (0.90–1.40)	1.25 (1.03–1.40)	0.421
LDL (mmol/L)	2.30 (1.80–3.00)	2.20 (1.60–2.98)	0.456
TG (mmol/L)	1.20 (0.80–1.70)	0.95 (0.63–1.60)	0.172
Endotoxin (EU/ml)	0.54 (0.37–0.73)	0.33 (0.26–0.41)	<0.001
LBP (μg/ml)	131 (104–165)	91 (76–110)	<0.001
sCD14 (μg/ml)	1.74 (1.35–2.25)	1.15 (1.04–1.41)	<0.001
LBP/sCD14 ratio	78.9 (65.4–91.6)	70.9 (60.0–95.4)	0.987
IL-8 (pg/ml)	8.92 (5.06–21.12)	4.69 (3.14–7.21)	<0.001
MCP-1 (pg/ml)	9.00 (4.88–16.75)	3.63 (2.22–7.09)	<0.001
CIMT (mm)	0.56 (0.50–0.64)	0.46 (0.42–0.51)	<0.001

MABP, mean arterial blood pressure; eGFR, estimated glomerular filtration rate (CKD-EPI); HDL, high density lipoprotein; LDL, low density lipoprotein; TG, triglyceride; LBP, lipopolysaccharide binding protein; sCD14, serum CD14I; IL-8, interleukin-8; MCP-1, monocyte chemoattractant protein-1. Result analyzed using Mann-Whitney test, with Chi-Square test for nonparametric data. Continuous data were expressed as mean ± SD or median (IQR) and categorical data as percentages.

### Endotoxin levels, inflammatory markers and CIMT

Endotoxin levels showed a positive correlation with sCD14 (r = 0.441, p<0.001) and MCP-1 (r = 0.388, p<0.001). Serum LBP showed a positive correlation with sCD14 (r = 0.605, p<0.001) and MCP-1 (r = 0.429, p<0.001). Carotid intima media thickness was also associated with MCP-1 (r = 0.448, p<0.001), sCD14 levels (r = 0.476, p<0.001), LBP (r = 0.340, p = 0.001), and IL-8 (r = 0.395, p<0.001). Eight patients had carotid plaques; and endotoxin levels were significantly higher among patients with carotid plaques compared to those without plaques (median 0.75 EU/ml; IQR 0.49–1.06 EU/ml versus median 0.53 EU/ml; IQR 0.33–0.71 EU/ml, p = 0.002).

### Atherosclerotic risk and inflammatory markers

Overall, circulating endotoxin levels were associated with increased risk of atherosclerosis [odds ratio: 4.95; 95% confidence interval: 2.52–9.73; p<0.001], with a more than four-fold increase in the risk of subclinical atherosclerosis among those with high endotoxin levels (˃ 0.5 EU/ml) compared with the reference group of patients with low endotoxin levels (≤ 0.5 EU/ml). Table 2 displays the results of the logistic regression analysis examining the association of subclinical atherosclerosis with combinations of endotoxin and IL-8 or MCP-1 or sCD14 stratum. In the fully adjusted model, patients with coexisting high endotoxin levels and high IL-8 levels or MCP-1 or sCD14 presented with significantly elevated risks of atherosclerosis. However, the risk of atherosclerosis in patients with high endotoxin levels was not influenced by the presence of high IL-8 or MCP-1 or sCD14 levels. Likewise, the risk of atherosclerosis in patients with low endotoxin levels was not influenced by exposure to high levels of IL-8 or MCP-1 or sCD14.

**Table 2:** Association of interleukin-8, monocyte chemoattractant protein-1 and serum CD14 levels with risk of early atherogenesis.

Categories	Serum concentration of immune mediators (Median; IQR)	Patients with subclinical atherosclerosis	Odds ratio (95% CI)	P value	Adjusted Odds ratio* (95% CI)	P value
Interleukin-8						
Endotoxin ≤ 0.5 EU/ml						
Interleukin-8 ≤ 7.99 pg/ml	4.8 (3.8–6.5)	14/36	1.0 (Reference group)		1.0 (Reference group)	
Interleukin-8 > 7.99 pg/ml	17.5 (10.5–38.8)	11/19	3.7 (1.0–13.7)	0.047	2.3 (0.5–11.1)	0.307
Endotoxin > 0.5 EU/ml						
Interleukin-8 ≤ 7.99 pg/ml	4.6 (3.6–6.5)	9/20	2.3 (0.7–7.4)	0.152	1.9 (0.5–7.4)	0.341
Interleukin-8 > 7.99 pg/ml	20.9 (13.4–122.8)	36/45	11.5 (4.3–31.2)	<0.001	8.5 (2.7–26.3)	<0.001
MCP-1						
Endotoxin ≤ 0.5 EU/ml						
MCP-1 ≤ 8.46 pg/ml	4.4 (2.1–6.6)	13/36	1.0 (Reference group)		1.0 (Reference group)	
MCP-1 > 8.46 pg/ml	15.1 (11.4–18.9)	12/19	6.0 (1.7–21.2)	0.007	3.4 (0.8–15.6)	0.113
Endotoxin > 0.5 EU/ml						
MCP-1 ≤ 8.46 pg/ml	5.8 (4.1–6.6)	9/21	3.0 (0.9–9.6)	0.064	2.3 (0.6–8.5)	0.213
MCP-1 > 8.46 pg/ml	15.6 (10.8–20.1)	36/44	15.6 (5.4–45.2)	<0.001	11.8 (3.5–40.5)	<0.001
Serum CD14						
Endotoxin ≤ 0.5 EU/ml						
Serum CD14 ≤ 1.7 μg/ml	1.2 (1.0–1.4)	16/39	1.0 (Reference group)		1.0 (Reference group)	
Serum CD14 > 1.7 μg/ml	2.1 (1.9–2.4)	9/16	2.2 (0.6–8.7)	0.250	1.3 (0.3–6.1)	0.730
Endotoxin > 0.5 EU/ml						
Serum CD14 ≤ 1.7 μg/ml	1.5 (1.1–1.6)	7/18	1.6 (0.5–4.9)	0.449	1.4 (0.4–4.9)	0.644
Serum CD14 > 1.7 μg/ml	2.2 (2.0–2.6)	38/47	10.9 (4.1–29.4)	<0.001	7.7 (2.6–23.4)	<0.001

Odds ratios, 95% confidence interval and p-values were derived from logistic regression analyses of subclinical atherosclerosis on endotoxin as well as interleukin-8, monocyte chemoattractant protein-1 and sCD14.

*Adjusted for Age, blood pressure, smoking, eGFR and cholesterol.

CIMT = carotid intima media thickness; CI = confidence interval, MCP-1 = monocyte chemoattractant protein-1

### TLR4 genotype distribution

The TLR4 Asp299Gly and Thr399Ile alleles were successfully genotyped in all, except for 4 of the samples in CKD patient group. Co-segregation of the Asp299Gly and Thr399Ile alleles was observed in 5 patients and 1 control, while 4 subjects (2 patients and 2 controls) had an isolated Asp299Gly polymorphism. The genotype distribution of wild-type TLR4 (AA genotype) did not show a significant difference among the patients and control subjects (93.9% vs 92.5%; P = 0.497). The occurrence of the G alleles was low (Table 3).

**Table 3:** Genotype and allele frequencies of TLR4 polymorphisms in patients and controls.

Genotype	Patients, N = 116^a^	Controls, N = 40	OR (95% CI)
**Asp299Gly**			
AA (wild-type)	109 (94%)	37 (92.5%)	
AG	7 (6.0%)	3 (7.5%)	
GG	0 (0%)	0 (0%)	
Frequency of the Gly allele, (%)	3.0	3.8	1.06 (0.70–1.62) p = 0.497
**Thr399Ile**			
CC (wild-type)	111 (95.7%)	39 (97.5%)	
CT	5 (4.3%)	1 (2.5%)	
TT	0 (0%)	0 (0%)	
Frequency of the Ile allele, (%)	2.2	1.3	0.89 (0.61–1.28) p = 0.513

OR = odds ratio; CI = confidence interval.

^a^ Four specimens were not genotyped in patient group

The heterozygous Asp299Gly (AG) allele was present in 7 patients and 3 controls, with minor allele frequency (MAF) of 3% and 3.8% respectively. The frequency of the variant genotype (CT) for TLR4 Thr399Ile allele was 4.3% in the patients and 2.5% in controls (MAF 2.2% and 1.3% respectively), whereas the CC genotype was prevalent in both the patient (96%) and control (98%) groups. The TLR4 Thr399Ile allele was present in only 6 of the genotyped subjects. The genotype of TLR4 polymorphisms in the studied population was in Hardy-Weinberg equilibrium (Asp299Gly: χ2 = 0.113; P = 0.736 for patients and χ2 = 0.061; p = 0.805 for controls; Thr399Ile: χ2 = 0.056; P = 0.812 for patients and χ2 = 0.006; P = 0.936 for controls). The GG and TT genotypes for Asp299Gly and Thr399Ile alleles, respectively, were not detected in the studied population.

### TLR4 polymorphisms, inflammatory markers and CIMT

Compared with the carriers of the wild-type TLR4, CKD patients with only the Asp299Gly allele and those with the combined Asp299Gly/Thr399Ile alleles had significantly lower serum levels of inflammatory markers and reduced CIMT values (Table 4). However, no association was observed between TLR4 polymorphisms and traditional risk factors for atherosclerosis.

**Table 4:** Relationship between TLR4 polymorphisms, traditional risk factors, CIMT and inflammatory markers in CKD patients.

Variables	Wild-Type TLR4 (N = 109)	TLR4 Asp299Gly+ Variant (N = 7)	TLR4 Asp299Gly+, Thr399Ile+ Variants (N = 5)	P-value	
				Wild-Type vs. Asp299Gly variant	Wild-Type vs. Combined TLR4 Variants Group
Age (Years); Mean ± SD	41.6 ± 10.0	40.1 ± 15.6	35.6 ± 16.4	0.894	0.253
Smoking (Yes/No)	15/94	2/5	2/3	0.285	0.109
Cholesterol (mmol/L)	4.20 (3.40–5.20)	4.40 (3.35–4.80)	4.40 (3.20–4.50)	0.958	0.972
HDL (mmol/L)	1.20 (0.90–1.40)	1.10 (0.95–1.30)	1.10 (1.00–1.30)	0.641	0.829
LDL (mmol/L)	2.30 (1.80–3.00)	2.70 (1.80–3.15)	2.70 (1.80–3.00)	0.655	0.668
TG (mmol/L)	1.20 (0.80–1.70)	1.10 (0.85–1.30)	1.00 (0.70–1.20)	0.475	0.225
MABP (mmHg)	143.0 (130.3–157.3)	137.0 (110.7–143.7)	137.0 (111.0–143.3)	0.181	0.350
Waist-Hip ratio	0.92 (0.87–0.96)	0.89 (0.84–0.92)	0.86 (0.81–0.89)	0.286	0.072
CIMT (mm)	0.57 (0.50–0.65)	0.49 (0.45–0.53)	0.49 (0.47–0.53)	0.009	0.028
IL-8 (pg/ml)	9.36 (5.58–22.33)	4.83 (2.90–6.26)	3.09 (2.70–4.83)	0.007	0.004
MCP-1 (pg/ml)	9.19 (5.12–17.03)	6.69 (2.87–7.45)	3.61 (2.12–7.67)	0.046	0.039
sCD14 (μg/ml)	1.76 (1.39–2.27)	1.30 (1.07–1.65)	1.30 (0.96–1.45)	0.047	0.054
LBP (μg/ml)	131.3 (108.4–165.9)	112.6 (76.1–118.7)	81.7 (70.5–113.6)	0.061	0.087

TLR4 = Toll-like receptor 4; HDL = High density lipoprotein; LDL = Low density lipoprotein; IL-8 = Interleukin-8; TG = Triglycerides; MABP = Mean arterial blood pressure; CIMT = Carotid intima media thickness; MCP-1 = Monocyte chemoattractant protein-1; sCD14 = Serum CD14; LBP = Lipopolysaccharide binding protein

## Discussion

We demonstrated that serum levels of inflammatory markers were markedly elevated in CKD patients compared with the controls, and positively correlated with CIMT, a surrogate marker of atherosclerosis. Furthermore, in support of previous studies that showed that LBP (marker of circulating endotoxaemia) was an independent predictor of atherosclerosis, this present study also established an association between elevated LBP levels and CIMT [35, 36]. Taken together, these findings indicate that low-grade inflammation is related to atherogenesis, and as previously reported may trigger a cascade of accelerated atherosclerosis in CKD patients [2].

In addition, we demonstrated that risk of atherosclerosis was associated with circulating endotoxaemia, and further analysis revealed that the risks were predicted by elevated levels of inflammatory markers suggesting that the severity of systemic inflammation plays a critical role in predicting the atherogenic potential of circulating endotoxaemia in CKD patients. This observation corroborated previous reports that identified increased levels of neopterin and soluble interleukin-2 receptors as independent predictors of vascular risk in a prospective population based study [37].

Even though our study is underpowered, we observed that the heterozygous Asp299Gly allele was associated with lower levels of inflammatory markers and reduced CIMT values, thus corroborating the findings of previous studies by Kiechl et al [16] and Ameziane et al [25]. On further analysis, our results suggest that the TLR4 polymorphisms seem to exact a major effect among patients with combined TLR4 Asp299Gly and Thr399Ile polymorphisms. Based on this observation, we hypothesize that TLR4 Asp299Gly and Thr399Ile polymorphisms may have a synergistic effect in our CKD patients.

Although the mechanisms by which TLR4 Asp299Gly and Thr399Ile polymorphisms mediate their synergistic effects are yet to be fully elucidated, newer evidence suggests that both the Asp299Gly and Thr399Ile mutations are within the fourth exon of the TLR4 gene, regulating a ligand-binding region and a coreceptor-binding region, respectively [38]. These two amino acid residues have been proposed to lie on the same side of the TLR4 molecule [39]. It has also been suggested that the Asp299Gly/Thr399Ile double mutant modifies an immunodominant epitope that, in turn, results in reduced expression of mutant TLR4 molecules [40]. Thus, it is possible that reduced expression of mutant TLR4 molecules, which might lead to attenuated TLR4 receptor signaling, could bring about the combined effect of TLR4 Asp299Gly and Thr399Ile polymorphisms observed in this study. Moreover, in TLR4 models, it has also been demonstrated that double mutant transfected cells showed a consistently greater hypo-responsiveness to low dose endotoxin than cells with either of the single polymorphic TLR4 variants [40]. It is also possible that individuals carrying both mutated genotypes of TLR4 alleles, which might be in linkage disequilibrium with mutations in the regulatory region of the TLR4 gene, could develop subnormal TLR4-mediated responses. Nevertheless, to elucidate the precise underlying mechanism would require further study on the stoichiometry, structure, and signaling of the TLR4/MD-2/CD14 complexes, which is beyond the scope of the present study.

The genotype frequencies of the Asp299Gly and Thr399Ile alleles observed in our study were similar to those reported by previous studies carried out in South African populations [41–43]. This study confirms and extends the findings of these previous studies, indicating that homozygote Asp299Gly and Thr399Ile polymorphisms are not common in the South African population.

Remarkably, we observed that only 6 of our cohorts had the heterozygous Thr399Ile polymorphism. This result further confirms the findings of Ferwerda et al. [44] who reported that the TLR4 Thr399Ile polymorphism is prevalent in the European population, but is almost in non-existent in the African populations, and this finding may help to explain our inability to demonstrate any association between the isolated Thr399Ile allele and a decreased risk of atherosclerosis in this study.

This study has its limitations: firstly, the descriptive and cross-sectional nature of this study does not allow for evaluation of cause-and-effect relationship between endotoxaemia-related inflammation and atherosclerosis. A prospective epidemiological study is needed to determine the contributory role of systemic inflammation on the risk of incident atherosclerosis in the South African CKD population. Secondly, our sample size was relatively small; a larger sample size comprising CKD populations from different ethnic groups would provide a more accurate reference database, given the spectrum of genetic diversity across the Southern African sub-region. The results of the TLR4 genotyping should therefore be confirmed in a larger CKD population from different ethnic groups to determine if the findings of this study are generalizable. Finally, our genotype analysis was restricted to the 2 common TLR4 SNPs. It is possible that we may have missed some novel polymorphisms that are unique to the South African population and would only be detected by whole genome sequencing analysis. Future studies aiming to sequence the whole genome using methods designed to assess variants that are specific for African populations would be a suitable alternative method for addressing this problem.

In conclusion, we demonstrated associations between circulating endotoxaemia, systemic inflammation and accelerated atherosclerosis among South African CKD patients. Our findings showed that the atherogenic predictive power of endotoxin was significantly increased by the presence of elevated serum levels of inflammatory mediators in South African CKD patients and that TLR4 polymorphisms are associated with low levels of inflammatory markers and CIMT values. These findings have two implications. First, endotoxin may function as a pro-inflammatory mediator of accelerated atherosclerosis in black South African CKD patients. Second, our findings provide support for a paradigm shift in the search for possible therapeutic targets to reduce atherosclerotic CVD in CKD patients. These might include prevention of endotoxaemia either through treating foci of endotoxin in CKD patients including periodontal disease, catheters and vascular access or by reducing translocation of endotoxin from the gut through reduction of gut venous congestion and/or oedema.
